# Molecular crosstalk between cancer cells and tumor microenvironment components suggests potential targets for new therapeutic approaches in mobile tongue cancer

**DOI:** 10.1002/cam4.24

**Published:** 2012-08-16

**Authors:** Dan Dayan, Tuula Salo, Sirpa Salo, Pia Nyberg, Sini Nurmenniemi, Daniela Elena Costea, Marilena Vered

**Affiliations:** 1 The Maurice and Gabriela Goldschleger School of Dental Medicine, Tel Aviv UniversityTel Aviv, Israel; 2 Department of Diagnostics and Oral Medicine, Institute of Dentistry, University of OuluOulu, Finland; 3 Oulu University HospitalOulu, Finland; 4 Institute of Dentistry, University of HelsinkiHelsinki, Finland; 5 Section for Pathology, The Gade Institute University of Bergen Haukeland HospitalBergen, Norway; 6 Institute of Pathology, The Chaim Sheba Medical CenterTel Hashomer, Israel

**Keywords:** Cancer-associated fibroblasts, mobile tongue cancer, molecular crosstalk, myoma model, protumorigenic inflammatory cells

## Abstract

We characterized tumor microenvironment (TME) components of mobile tongue (MT) cancer patients in terms of overall inflammatory infiltrate, focusing on the protumorigenic/anti-inflammatory phenotypes and on cancer-associated fibroblasts (CAFs) in order to determine their interrelations and associations with clinical outcomes. In addition, by culturing tongue carcinoma cells (HSC-3) on a three-dimensional myoma organotypic model that mimics TME, we attempted to investigate the possible existence of a molecular crosstalk between cancer cells and TME components. Analysis of 64 cases of MT cancer patients revealed that the overall density of the inflammatory infiltrate was inversely correlated to the density of CAFs (*P* = 0.01), but that the cumulative density of the protumorigenic/anti-inflammatory phenotypes, including regulatory T cells (Tregs, Foxp3+), tumor-associated macrophages (TAM2, CD163+), and potentially Tregs-inducing immune cells (CD80+), was directly correlated with the density of CAFs (*P* = 0.01). The hazard ratio (HR) for recurrence in a TME rich in CD163+ Foxp3+ CD80+ was 2.9 (95% CI 1.03–8.6, *P* = 0.043 compared with low in CD163+ Foxp3+ CD80+). The HR for recurrence in a TME rich in CAFs was 4.1 (95% confidence interval [CI] 1.3–12.8, *P* = 0.012 compared with low in CAFs). In vitro studies showed cancer-derived exosomes, epithelial–mesenchymal transition process, fibroblast-to-CAF-like cell transdifferentiation, and reciprocal interrelations between different cytokines suggesting the presence of molecular crosstalk between cancer cells and TME components. Collectively, these results highlighted the emerging need of new therapies targeting this crosstalk between the cancer cells and TME components in MT cancer.

## Introduction

Over 400,000 new cases of oral cancer are expected to be diagnosed worldwide each year, and >50% are cancers of the mobile/oral tongue (MT) [1, 2]. Despite advances in surgery, radiotherapy, and chemotherapy treatment, the mortality rate has remained essentially unchanged for the last four decades, with a 5-year survival rate of around 50% [3]. The incidence of MT cancer is currently reported to be increasing compared with other head and neck sites, especially in the 20- to 44-year age group [4, 5]. We recently showed that components of the tumor microenvironment (TME) have a cardinal role in the molecular biology of MT cancer in terms of the invasion and spread of the tumors, with a direct impact on patients' clinical outcomes [5]. Specifically, we found that the cancer-associated fibroblasts (CAFs) have a negative impact on overall and specific survival [6, 7]. In addition, we provided evidence that CAFs were present in the metastatic lymph nodes similarly to matched primary tongue tumors, thus suggesting that CAFs not only promote tumor invasion but also facilitate metastasis [8].

The inflammatory infiltrate of a TME is an essential source of cytokines, chemokines, growth factors, angiogenic factors, and enzymes [9–11]. These are produced and released in considerable amounts by certain types of inflammatory cells that were shown to be implicated in tumor growth, progression, and invasion in human colon and breast cancer [9, 10]. As such, these protumorigenic inflammatory cells comprise “bad inflammation,” in contrast to components of the inflammatory infiltrate that represent an antitumorigenic force and, therefore, constitute “good inflammation” [9]. Furthermore, the molecular interactions between the inflammatory infiltrate and other components of TME (e.g., CAFs) are assumed to be of special importance in view of the finding that CAFs can express inflammation-related genes and corresponding protein products [12, 13]. In oral cancer, the general notion is that the more abundant the inflammatory infiltrate surrounding the tumor, the better the prognosis of the patients [14, 15], but there has been very little in-depth analysis of the inflammatory infiltrate. One recent study associated the frequency of the regulatory T cells with a lower grade of histological differentiation [16]. In another study in which cases of squamous cell carcinoma (SCC) of the oral cavity were analyzed together with cancers of the oropharynx, hypopharynx, epilarynx, and larynx, it was reported that regulatory T cells (bearing a Foxp3+ CD4+ phenotype) were positively correlated with locoregional control [17]. The newly recognized differences in etiology, epidemiology, and clinical outcomes of the tumors in the oral cavity compared with those of the oropharynx and larynx called for re-evaluation of previously published conclusions [17]. Moreover, a very recent study on oral SCC cases demonstrated a positive correlation between tumor-associated macrophages (TAM) of the M2 (TAM2) phenotype and CAFs and poor prognosis [18]. However, no study has specifically examined the overall inflammatory infiltrate in MT cancer or the constituent inflammatory cell types, or looked into how this inflammatory infiltrate relates to CAFs and to clinical outcomes.

It was the aim of this study to delineate the profile of the inflammatory infiltrate in a TME of MT cancer, with emphasis on the protumorigenic components, and to assess their interrelations with CAFs. We also analyzed how all these elements were associated with clinical outcomes. In addition, using a new in vitro three-dimensional myoma organotypic model [19], we investigated the possible occurrence of pathways of molecular crosstalk between cancer cells and various TME components in order to provide new candidate molecular therapeutic targets.

## Materials and Methods

### MT cancer patients

Sections from resection specimens of a total 64 patients with MT cancer were used in this study, 31 females (mean age 65 ± 11.7 years) and 33 males (mean age 57.4 ± 17.9 years). The patients were staged according to the 1997 International Union Against Cancer classification (TNM), where stages I and II were considered early-stage (*n* = 15) patients and stages III and IV as late-stage (*n* = 49) patients. None of the patients was previously treated, all had undergone surgery, and complementary radiotherapy (*n* = 32), radio- and chemotherapy (*n* = 10), or only chemotherapy (*n* = 1) was administered when indicated. All patients were treated at the Chaim Sheba Medical Center, Tel Hashomer, Israel, between 1990 and 2006. The study was approved by the IRB of the medical center. Ten patients with no evidence of disease (NED) who were followed for less than 18 months were deleted from the survival analysis. For the remaining 54 patients, the mean follow-up was 63 ± 43 months. The clinical outcomes were measured by two endpoints: locoregional disease control expressed by locoregional recurrence (LR) and overall survival (OS). Time to recurrence was calculated as the interval between the date of diagnosis and the first sign of treatment failure at the primary tumor site, at the site of cervical metastases, or both. The OS calculation included patients alive and free of disease and those alive with disease at the last follow-up visit.

### TME components in the specimen sections

#### Inflammatory infiltrate

##### Morphometrical density (hematoxylin and eosin–stained slides)

Assessment was performed throughout the tumor section at the TME interface in each case and it was classified as 1 = absent or limited, 2 = dense but intermittent, and 3 = dense and continuous infiltrate around the tumor.

##### Immunohistochemistry and immunomorphometry of inflammatory cells and nuclear factor kappa-B

The antibodies used to identify the various classes of inflammatory cells and nuclear factor kappa-B (NF-κB) and the essentials of preparations of the immunostains are included in the supplemental material. The percentage of the positively stained cells from the entire population of inflammatory cells was assessed for each staining in each case. Positive staining of the CD80+ cells included the inflammatory and spindle-shaped CAF-like cells within the TME. Similar to the CD80+ cells, positive staining of CD163 cells included the inflammatory spindle-shaped CAF-like cells and the endothelial cells within the TME. For survival analysis, each of the results (presented as percentages of positively stained cells) was classified into “low” and “high” groups, with the median value considered the cutoff point (i.e., low ≤ median and high > median). In addition, we further converted the results expressed as percentages to a scoring system (described below).

Foxp3-stained cells were relatively sparse and were therefore scored semi-quantitatively on a scale from 0 to 4: 0 = no stained cells, 1 = a few dispersed cells, 2 = similar to “1” with the addition of small foci consisting of <10 cells, and 3 = similar to “2” with foci comprising >10 cells. For survival analysis, Foxp3 was grouped into low score (scores of 0, 1, and 2) versus a high score (a score of 3).

The results that were expressed as percentages of the positively stained inflammatory cells were converted to the following scoring system for comparisons in the statistical analysis: 1 = ≤10% positive cells, 2 = 11–25% positively stained cells, 3 = 26–75% positive cells, and 4 = >75% positively stained cells. Using this system, and being interested in detecting possible associations between the additive impacts of the TME cells with an anti-inflammatory/protumorigenic function and the clinical outcomes, we combined the scores of CD80+ Foxp3+ CD163+. In this way, we were able to arrive at the cumulative score of the cells that represented the unified anti-inflammatory/protumorigenic forces within the TME of MT cancer. The results of the scoring system for each individual stain as well as for the cumulative scores were expressed as mean (±SD) and median scores. For purposes of statistical analyses, the median score served as the cutoff point, where mean scores equal to or less than the median were considered low and those higher than the median as high.

NF-κB staining was assessed as the percent of positive inflammatory cells from the total of inflammatory cells. NF-κB was considered being positive when it was identified in both the nuclear and cytoplasmic cellular compartments due to the occurrence of cytoplasmic-nuclear shuttling [20].

Appropriate positive and negative controls were applied for all the described immunohistochemical procedures.

#### Cancer-associated fibroblasts

CAFs, as identified by α-smooth muscle actin (*α*-SMA), were assessed as previously described [6] using a 5-scale scoring system: 0 = absent, 0.5 = a few spindle-shaped CAFs at the periphery of the SCC, 1 = CAFs surrounding the tumor in a few concentric layers in several foci, 2 = CAFs with both spindle-shaped and plump morphology in many areas of the tumor, and 3 = similar to “2” but CAFs exceptionally abundant throughout the section, occasionally exceeding the carcinomatous component [6, 7]. For statistical survival analysis, the *α*-SMA-stained CAFs were classified into CAF poor-to-intermediate (scores of 0, 0.5, 1, and 2) versus CAF-rich (a score of 3) groups.

#### Staining of the tumor cells

In addition, the percentages of the CD80+ and the NF-κB+ tumor cells from the entire tumor mass were assessed.

### In vitro three-dimensional myoma organotypic model

Tongue SCC cells were cultured on the disks of an in vitro three-dimensional myoma organotypic model as previously described [19]. The reason for using this model was its advantage in approximating the natural TME in both the variety of the cellular components and the presence of various extracellular matrix proteins and glycoproteins [19]. Briefly, uterine leiomyoma tissue was retrieved from routine surgical operations after obtaining informed consent of the donors. (The study was approved by the Ethics Committee of the Oulu University Hospital, Oulu, Finland.) Four-millimeter-thick, 8-mm-diameter myoma disks were prepared and stored at −70°C in cell culture media with 10% fetal bovine serum (FBS). To prepare myoma-based organotypic cultures, myoma disks were equilibrated in culture medium at room temperature for 1 h, after which cancer cells were added on top of each disk. Human tongue SCC cells HSC-3 (JCRB 0623; Osaka National Institute of Health Sciences, Osaka, Japan) were cultured in 1:1 Dulbecco's modified Eagle's medium (DMEM)/F-12 (Invitrogen, Carlsbad, CA) supplemented with 100 U/mL penicillin, 100 g/mL streptomycin, 50 g/mL ascorbic acid, 250 ng/mL fungizone, 5 *μ*g/mL insulin (bovine pancreas), 0.4 ng/mL hydrocortisone (all reagents from Sigma-Aldrich, St. Louis, MO, USA ), and 10% heat-inactivated FBS St. Louis, MO, USA (Invitrogen, Carlsbad, CA, USA).

Some assays were performed using also human gingival fibroblasts (GF) which were obtained from biopsies of healthy gingiva [21]. They were cultured in DMEM supplemented with 100 U/mL penicillin, 100 g/mL strepto-mycin, 50 *μ*g/mL ascorbic acid, 250 ng/mL fungizone, 1 mmol/L sodium pyruvate, and 10% heat-inactivated FBS. Other assays included also carcinoma-associated fibroblasts derived from a specimen of tongue SCC. The explants that had an outgrowth of cells with fibroblastic (elongated) morphology were selected and isolated with clonal rings, and the outgrowth of cells was trypsinized and subsequently cultured in fibroblast routine culture medium (same as above). These cells, named CaDEC12, were characterized at passage 3 for the expression of lineage-specific markers by flow cytometry and immunohistochemistry. They expressed the fibroblast markers vimentin (98.67% positive cells) and platelet-derived growth factor receptor (PDGFR)-B (97.60% cells) and were negative for epithelial (epidermal surface antigen [ESA] 0.09% positive cells), endothelial (CD31 0.65% positive cells), and hematopoietic (CD45 0.25% positive cells) lineage-specific markers. Very low levels of the pericyte/mesenchymal stem cell marker CD146 (1.48% positive cells) were detected. In addition, *TP*53 (exons 5–9) mutation analysis had been performed using genomic DNA, and it revealed that CaDEC12 fibroblastic cells were harboring wt p53 while the epithelial cells derived from the same tumor showed a p53 mutation in exon 7 (codon 772 G>A).

In all monoculture assays (HSC-3 or GF), 3–4 × 10^5^ cells were cultured on the top of myoma tissue for 14 days. In the coculture assays, 2–3 × 10^5^ HSC-3 cells were first mixed with 4 × 10^5^ GFs or CaDEC12 cells and then cultured on myoma tissue for 14 days.

The same assays of mono- and cocultures that had been performed on the myoma model were also performed on rat type I collagen gel for an identical period of time which served as controls, as previously described [19].

All experiments of the myoma and collagen assays are listed in Table 1.

**Table 1:** Antibodies for immunostains of the in vitro three-dimensional myoma and collagen gel assays

Investigated pathways	Markers	Clone, working procedure	Manufacturer
Exosomal markers	TGF-*β*	Polyclonal, 1:25, overnight, citrate buffer pH 6, pressure cooker	Acris, Herford, Germany
	TSG 101	Polyclonal, 1:30, overnight, EDTA pH 9, pressure cooker	Proteintech Group, Chicago, IL
CAFs	*α*-SMA	Clone 1A4, 1:100, 60 min; citrate buffer, pH 6, microwave at 92°C	Dako A/S, Denmark
Production and maintenance of inflammation	IL-1a	Polyclonal, 1:100, overnight, no antigen retrieval procedure needed	Acris, Herford, Germany
	IL-8	Polyclonal, 1:50, overnight, no antigen retrieval procedure needed	Proteintech Group, Chicago, IL
	IL-1R1 (CD121A)	Polyclonal, 1:50, overnight, EDTA pH 9, pressure cooker	Acris, Herford, Germany
	NF-kB	Polyclonal, NF-kB/p65 1:500, overnight, citrate buffer pH 6, pressure cooker	Alexis Biochemical San Diego, CA
	CXCL12 (SDF-1*α*)	Clone 79018, 1:50, overnight, EDTA pH 9, pressure cooker	R&D Systems, Minneapolis, MN
	CXCR4	Clone 44716, 1:50, overnight, EDTA pH 9, pressure cooker	R&D Systems, Minneapolis, MN
	CD80	Clone 1G10, 1:50, overnight, citrate buffer pH 6, pressure cooker	Abcam, Cambridge, U.K.
	Foxp3	Clone mAbcam 22510, 1:50, overnight, EDTA pH 9, pressure cooker	Abcam, Cambridge, U.K.
EMT (double-stain)	Pan-cytokeratin (DAB)	AE1/AE3 (mouse), 1:50, overnight, citrate buffer pH 6, pressure cooker	Zymed/Invitrogen, Carlsbad, CA
		–*Color reaction: DAB, 10 min*	
	Twist (fast red)	Polyclonal (rabbit), 1:50, overnight, citrate buffer pH 6, pressure cooker	Zytomed, Berlin, Germany
		–*Color reaction: permanent AP-red chromagen, 10 min*	
		Secondary antibody: HRP-AP rabbit, 45 min, room temperature	Innovex Biosciences, Richmond, CA

CAF, cancer-associated fibroblasts; EMT, epithelial–mesenchymal transition; TGF-*β*, transforming growth factor-*β*; IL, interleukin; NF-kB, nuclear factor kappa-B; *α*-SMA, alpha smooth muscle actin; DAB, 3,3-diamino benzidine.

#### Immunohistochemical staining

Serial sections were routinely processed as previously described for the human sections. At the end of the culture, the organotypic and collagen culture specimens were fixed overnight in 4% neutral-buffered formalin and embedded in paraffin. Six-micron-thick sections were deparaffinized and immunostained with various antibodies, as detailed in Table 1. For double immunostaining, the sections were exposed simultaneously to both primary antibodies and then to the second antibody, followed by staining with DAB for identification of the cytokeratin reaction (brown color) and by AP-red chromogen (red–purple color) for the identification of twist reaction.

TSG101 was used to identify tumor-derived exosomes, known to carry signals to induce their transdifferentiation of stromal fibroblasts into CAFs [22]. One of these signals was transforming growth factor-*β* (TGF-*β*) [23]. *α*-SMA was used to identify possible formation of CAF-like cells around the HSC-3 clusters.

Twist, a key marker of the epithelial–mesenchymal transition (EMT) process [8], is assumed to be involved in the emergence of CAF-like cells from carcinoma cells. Therefore, we attempted to identify HSC-3 cells positive either for both cytokeratin and twist or HSC-3 cells positive only for twist, implying loss of epithelial differentiation.

Interleukin-1a (IL-1a), IL-1R1, IL-8, and NF-κB are all known to have reciprocal interactions. IL-1 can induce expression of NF-κB through IL-8, NF-κB induces expression of IL-1a, and interleukin-1 receptor 1 (IL-1R1) serves as the receptor through which NF-κB activates its downward cascade of intracellular effects [24]. CXCL12/SDF-1 (produced mainly by inflammatory cells) and its receptor, CXCR4 (expressed mainly by the tumor cells), were selected for their role in tumor migration and invasion [25]. Two sets of assays were examined for each monoculture or coculture of the myoma model and controls.

#### Immunomorphometry

Assessment of the immunostains was performed semi-quantitatively on a scale of 0 to 5, where 0 = no staining, 1 = staining of weak intensity in <50% cells, 2 = weak but extensive (>50% cells) staining, 3 = strong staining in <50% cells, and 4 = strong staining in >50% cells. The HSC-3 and, whenever applicable, the myoma cells or fibroblasts in the control assays were assessed. This semi-quantitative assessment attempted to give a general overview of the staining patterns in each type of in vitro assay and, as such, was not further processed by statistical analysis. The results of double immunostaining of the sections by twist and cytokeratin were solely described qualitatively.

### Statistical analysis

Statistical tests included Pearson's correlations crosstabs and survival analysis.

Univariate analysis was performed using the Kaplan–Meier method, and significance was confirmed by the log-rank test. Multivariate analysis was done using the Cox's proportional hazards regression model by forward variable selection that employed likelihood ratio criteria and included those covariates that showed significant associations with recurrence or survival by the univariate analysis. All statistical analyses were done using SPSS, version 15 (SPSS Inc., Chicago, IL), and the significance level was set at *P* < 0.05.

## Results

### Human sections of MT cancer

#### Morphometrical density of the inflammatory cells

**Figure 1:**
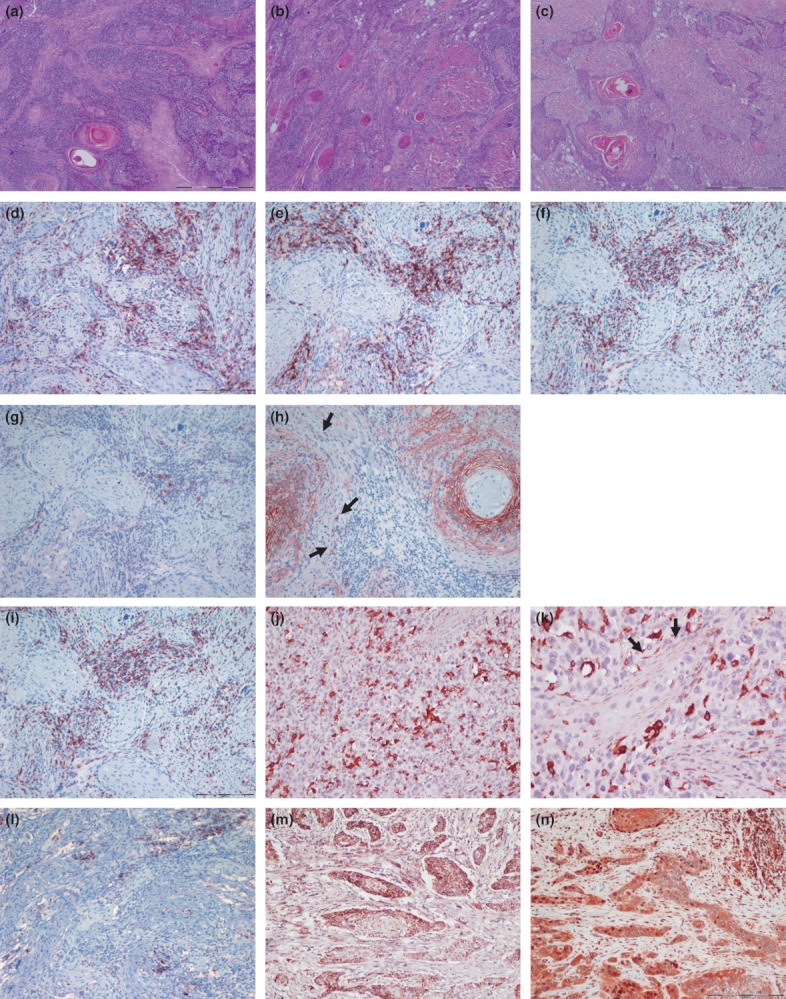
(a) Density of the inflammatory infiltrate with a dense and continuous pattern, (b) a dense but discontinuous pattern, and (c) a lack of inflammatory infiltrate (hematoxylin and eosin, original magnification 40×). (d) Samples of specific types of inflammatory cells comprised pan T lymphocytes, (e) CD4 lymphocytes, (f) CD8 lymphocytes, (g) B lymphocytes, (h) plasma cells, arrows (positive immunostaining also seen in the tumor islands), (i) CD68 macrophages, (j) CD163 macrophages, (k) CD163 stromal cells (arrows), (l) regulatory T cells (Foxp3-positive), and (m) CD80-positive cells. Nuclear NF-kB was present in both the tumor cells and the inflammatory cells (n). Photomicrographs (d–n) were at 100× original magnification with the exception of (m), which was at 200×.

The most frequently encountered pattern of inflammatory infiltrate was dense but noncontinuous (35 cases, 54.7%) followed by limited-to-absent (17 cases, 26.5%) and then dense and continuous (12 cases, 18.8%) (Fig. 1a–c).

#### Types of inflammatory cells: frequency and correlations

The pan T-cell lymphocytes as a group comprised about half of the inflammatory infiltrate (54 ± 13%). When analyzed with respect to separate T-cell phenotypes, CD4+ cells (36.5 ± 14%) were more abundant than CD8+ cells (18 ± 15%). Mature macrophages of the CD68+ phenotype (47 ± 27%) were almost as frequent as the T cells. Plasma cells (33 ± 16%) and B-cell lymphocytes (26 ± 14%) were less common than the pan T cells and macrophages. Cells with a CD163+ phenotype were frequent (40 ± 26%), and those with a CD80+ phenotype were abundant (77 ± 16%). High-Foxp3 cases (77.8%) were more common than the low-Foxp3 cases (32.2%). It should be noted that the sum of percentages of the various immune cells may be higher than 100% as there are cells that can express markers characteristic to more than one cell phenotype; for example, B cells may also be CD138 positive [26] and macrophages may be CD4 positive (Fig. 1d–m) [27].

NF-κB expression (Fig. 1n) was frequent in the inflammatory cells (85 ± 8%), and it was inversely correlated with the frequency of the pan T cells (*P* = 0.003, *r* = −0.45), the CD8+ cells (*P* = 0.005, *r* = −0.45), the CD4+ cells (*P* = 0.041, *r* = −0.32), and the plasma cells (*P* = 0.025, *r* = −0.35).

#### CAFs: frequency and correlations

Cases with a low-to-intermediate density of CAFs (*n* = 35, 68.6%) were more frequent than CAF-rich cases (*n* = 16, 31.4%). CAF density was inversely correlated with the density of the inflammatory infiltrate (*P* = 0.01) and positively correlated with the cumulative CD163+ CD80+ Foxp3+ score (*P* = 0.01).

#### Immunoexpression of inflammatory markers by the tumor cells

Expression of CD80 was abundant on the tumor cells (82 ± 13%). NF-κB expression was also frequent in the tumors (87 ± 7%). The expression of NF-κB in the inflammatory cells was positively correlated with that within the tumor (*P* < 0.001, *r* = 0.55)

#### Protumorigenic inflammatory infiltrate and CAFs: associations with clinical outcomes

**Figure 2:**
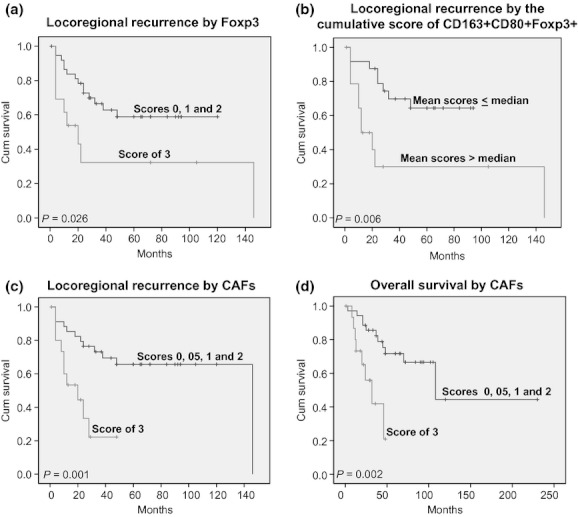
(a) Kaplan–Meier analysis for locoregional recurrence by Foxp3. (b) Kaplan–Meier analysis for locoregional recurrence by the cumulative score of CD163+ CD80+ Foxp3+. (c) Kaplan–Meier analysis for locoregional recurrence by CAF. (d) Kaplan–Meier analysis for overall survival by CAF scores.

Univariate analysis demonstrated that a high Foxp3 score had a negative impact on recurrence (*P* = 0.026) (Fig. 2a), while the density of the inflammatory infiltrate as well as the other individual types of inflammatory cells and the expression of NF-κB had no impact on either recurrence or patient survival (*P* > 0.05). The cumulative high CD163+ CD80+ Foxp3+ score had a negative influence on recurrence (*P* = 0.006) (Fig. 2b). A CAF-rich score had a negative impact on recurrence (*P* = 0.001) (Fig. 2c) and was associated with poor survival (*P* = 0.002) (Fig. 2d).

Multivariate analysis of the associations between CAFs and the inflammatory protumorigenic inflammatory components and clinical outcomes included parameters of CAFs, Foxp3, and the cumulative CD163+ CD80+ Foxp3+ score. The results for recurrence showed that CAF-rich cases had a hazard ratio (HR) of 4.1 (95% confidence interval [CI] 1.3–12.8, *P* = 0.012) compared with CAF poor-to-intermediate; high CD163+ CD80+ Foxp3+ score had an HR of 2.9 (95% CI 1.03–8.6, *P* = 0.043) versus low CD163+ CD80+ Foxp3+ score. The results for survival showed that the CAF-rich score had an HR of 6.7 (95% CI 1.9–23.7, *P* = 0.003) compared with the CAF poor-to-intermediate score.

### In vitro three-dimensional myoma organotypic model

**Figure 3:**
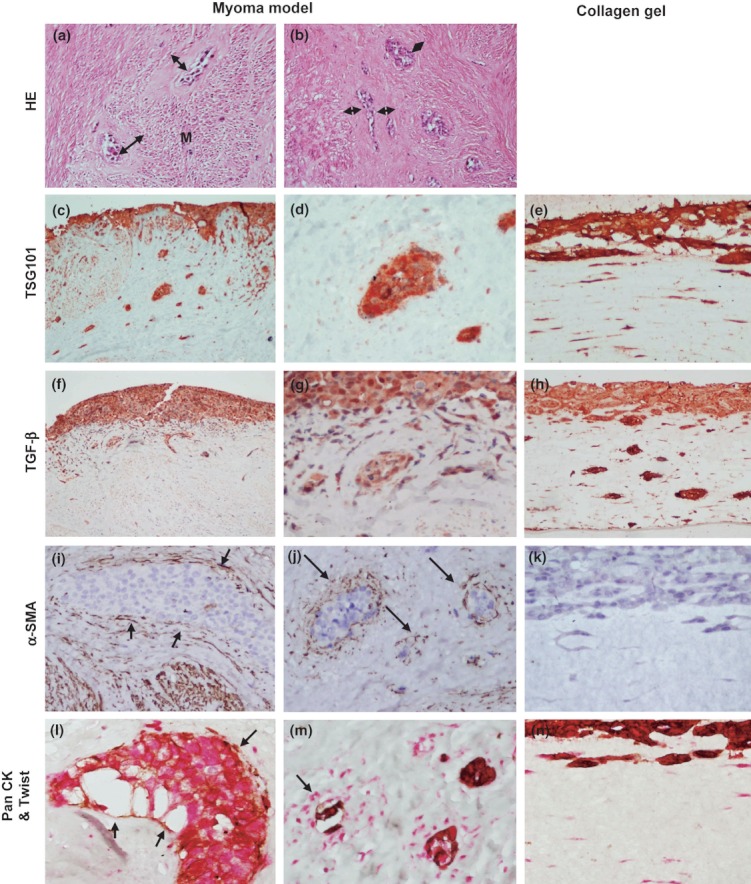
(a and b) Myoma model and collagen assay. Hematoxylin and eosin stain shows a collagenous “collar-like” area that surrounds the tumor islands (arrows) that invade into the smooth muscle tissue of the myoma (M) (original magnification 200×). (c) TSG101 in the myoma model shows a strong and diffuse staining of the superficial lining and deeply invading tumor islands/clusters (original magnification 100×). (d) The stromal cells surrounding the tumor island are also TSG101 positive when seen at a higher magnification (original magnification 400×). (e) Both the HSC-3 cells and fibroblasts are TSG101 positive in the collagen assay (original magnification 100×). (f and g) TGF-*β* immunostain in the myoma assay (original magnification 100× and original magnification 400×, respectively) and (h) in the collagen assay (original magnification 100×) exhibit a similar pattern of expression as TSG101. (*i*) *α*-SMA staining in a sample of monoculture: a tumor island is surrounded at its periphery by delicate, spindle-shaped *α*-SMA-positive cells (arrows). (j) A sample of coculture of HSC-3 cells and CaDEC cells: small invading tumor islands (arrows) are closely surrounded by concentric layers of *α*-SMA-positive cells. (k) No *α*-SMA-positive cells were found in the collagen assay (i, j, and k, original magnification 400×). (l) Double immunostaining with pan-cytokeratin (CK) (brown) and twist (red–purple): the same field as illustrated in (i), shows that those *α*-SMA-positive cells are both pan-CK and twist positive. (m) The same field as illustrated in (j) shows that those cells positive for *α*-SMA are twist positive. (n) No stromal cells were seen at the tumor–stroma interface in the collagen assay, as was the case for (k). (l, m, and n, original magnification 400×).

As previously described, all carcinomas cultured on top of myomas generally exhibited invasion of varying depths into the smooth muscle tissue mass, while those cultured over collagen gels were only minimally invasive [19]. Notably, tumor islands that invaded into the muscle bundles were usually separated from them by an acellular, collar-like, collagenized area (Fig. 3a and b; Table 2).

**Table 2:** In vitro immunoreactivity of the HSC-3 cell line and the stromal components

	TGS101		TGF-*β*		*α*-SMA	IL-1a		IL-8		NF-kB		CXCR4		CXCL12		CD80		Foxp3	
										
	T	F/M	T	F/M	F/M	T	F/M	T	F/M	T	F/M	T	F/M	T	F/M	T	F/M	T	F/M
Myoma		2		1	*		0		2nc		2		4		0		0		0
		4		1	*		0		2nc		1		4		0		0		0
Myoma + GF		2		1	*		−		2nc		3		4		0		0		0
		4		2	*		0		−		3		4		0		0		0
Myoma + HSC-3	4	4	4	1	*, CAF	1nc	0	4cn	2nc	4	2	4	4	0	0	4	0	0	0
	4	2	4	2	*, CAF	4nc	0	2cn	2cn	4	2	4	4	0	0	3	0	0	0
Myoma + HSC-3 + GF	2	1	4	2	*	4nc	0	4nc	2c	4	1	4	4	0	0	4	−	0	−
	2	2	2	1	*	4nc	0	4cn	4nc	4	2	2	4	0	0	4	0	0	0
Myoma + HSC-3 + CaDEC	4	2	4	2	*, CAF	4nc	0	4cn	2cn	4	2	4	4	1	0	1	0	1	0
	2	2	2	1	*, CAF	4nc	0	4cn	2c	4	2	4	4	1	0	2	0	0	0
Collagen + GF		4		4	0		2c		4nc		4		4		0		0		0
		4		2	0		4c		4cn		4		4		1		3		2
Collagen + HSC-3 + GF	4	−	4	−	0	4c	4nc	4cn	4cn	4	−	4	−	0	0	2	−	1	1
	4	4	4	4	0	4c	4c	4n	4c	2	1	4	4	0	0	1	1	1	1
Collagen + HSC-3 + GF	4	4	4	4	0	4c	4cn	4cn	4cn	4	4	4	4	0	0	2	2	2	2
	4	4	4	4	0	4c	4c	4cn	4cn	4	4	2	2	0	0	4	3	2	2

The numbers refer to the scoring system (0 = no staining, 1 = staining of weak intensity in <50% cells, 2 = weak but extensive [>50% cells] staining, 3 = strong staining in <50% cells, and 4 = strong staining in >50% cells).

IL-1R1 (CD121A) staining was positive in all examined sections with a score of 4 (see text) in the HSC-3 cells as well as in the fibroblasts/myoma cells and therefore was not included in this table.

T, tumor; F/M, fibroblasts/myoma cells; GF, gingival fibroblasts; CaDEC12, CAF cell line; c, cytoplasmic staining; n, nuclear staining; cn, cytoplasmic staining more frequent than nuclear staining; nc, nuclear staining more frequent than cytoplasmic staining; (−), no staining; *, the myoma smooth muscle cells were positive.

HSC-3 cells were extensively (>50%) stained for TSG101 in the myoma monocultures and cocultures and the staining was of strong-to-weak intensity (Fig. 3c and d). Similar to TSG101, HSC-3 cells were diffusely and usually strongly positive for TGF-*β* (Fig. 3f and g). The myoma cells and other microenvironmental cells were diffusely stained, but usually at a weaker intensity than that of the HSC-3 cells for both TSG101 and TGF-*β*. Both HSC-3 cells and fibroblasts yielded an extensive and strong staining for TSG101 and TGF-*β* in the collagen cultures (Fig. 3e and h).

Special attention was paid to the immediate periphery of the HSC-3 cells when examining *α*-SMA staining in the myoma assays. The SMA-positive, delicate, spindle-shaped cells surrounding the HSC-3 infiltrating nests/clusters were found exclusively in the cocultures of myoma and HSC-3 cells and in those of myoma, HSC-3, and CaDEC12 cells. The *α*-SMA-positive cells were arranged in one or more concentric layers closely around the tumor (Fig. 3i and j). This pattern was similar to the CAF and tumor interrelations seen in the human sections. Using double immunostaining with pan-cytokeratin and twist, we found that the HSC-3 cells cultured on top of myomas exhibited only partial cytokeratin immunoreactivity (brown color), while the invasive islands were predominantly negative, with only occasional remnants of cytokeratin. Twist expression was occasionally observed in HSC-3 cell layers on top of myomas (purple–red color), and it was predominant within the invading tumor islands concomitantly with the disappearance of cytokeratin. Interestingly, in sections and locations with *α*-SMA-positive CAF-like cells, we also identified delicate, spindle-shaped cells at the periphery of the invading tumor islands or closely surrounding them that were either positive to both cytokeratin and twist in the HSC-3-myoma monocultures or, alternatively, only to twist in the HSC-3-CaDEC12-myoma cocultures (Fig. 3l–m). No *α*-SMA-stained cells were detected in the collagen cultures. The HSC-3 cells were strongly positive for cytokeratin and twist in these assays (Fig. 3n). Even some downward anatomizing extensions of the HSC-3 cells retained a strong double staining with cytokeratin and twist and were not found to be only twist positive, unlike in the myoma model.

**Figure 4:**
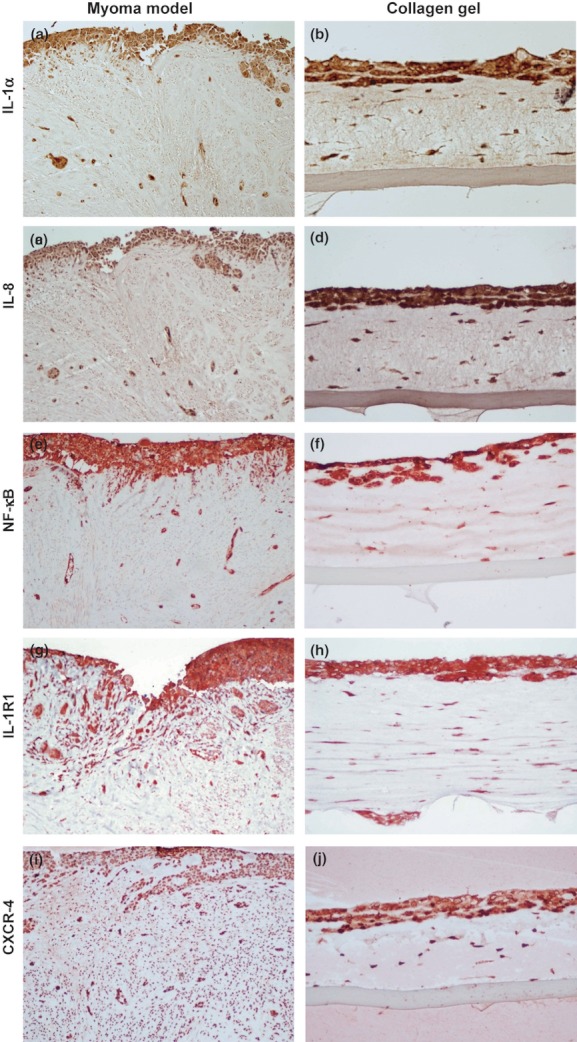
Immunohistochemical stains of the myoma model and collagen assay for IL-1a (a and b), IL-8 (c and d), NF-kB (e and f), IL-1R1 (g and h), and CXCR4 (i and j) (a, c, e, g, and i, original magnification 40×; b, d, f, h, and j, original magnification 100×).

The cytokines IL-1a and IL-8 usually exhibited diffuse and strong expression in the HSC-3 cells (partly also exhibiting a nuclear location) in the myoma assays. IL-1a was negative and IL-8 was diffusely and weakly positive in the surrounding stromal cells. NF-κB, the transcription factor that has a reciprocal relation with the expression of the IL-8 gene and its protein product [25, 28, 29], was diffusely and strongly positive in the HSC-3 cells and less so in the myoma and other stromal cells. In addition, the IL-1 receptor, IL-1R1, was diffusely and strongly positive in the HSC-3 cells and in the myoma and other stromal cells. Both the HSC-3 cells and fibroblasts were diffusely and strongly positive for IL-1a, IL-8, IL-1R1, and NF-κB in the collagen assays. Examples of the immunostaining pattern of the examined cytokines are illustrated in Figure 4.

CXCR4 in both the myoma model and the collagen assay was usually diffusely and strongly positive in both the HSC-3 and stromal cells. CXCL12, the CXCR4 ligand, CD80 and Foxp3 were either absent or had a limited expression in both the myoma and collagen assays in the HSC-3 cells as well as in the other types of surrounding cells.

## Discussion

The uniqueness of this study lies in its multifactorial analysis of the TME components in MT cancer that encompass both the inflammatory infiltrate and CAFs. We investigated the density of the overall tumor-associated inflammatory infiltrate and examined a wide panel of different types of inflammatory cells with special emphasis on those with an anti-inflammatory/protumorigenic phenotype. Furthermore, we analyzed the relationship between the inflammatory infiltrate and CAFs and their correlations with clinical outcomes.

The present results supported those of our previous studies which showed that a high frequency of CAFs in MT cancer was associated with poor clinical outcomes [6, 7]. We also found that a high frequency of CAFs was inversely correlated to the extent of the overall inflammatory infiltrate, suggesting that, unlike CAFs, the inflammatory infiltrate is positively associated with clinical outcomes, in agreement with the results of other studies [14, 15]. However, examination of known protumorigenic components of the inflammatory infiltrate, such as regulatory T cells (Foxp3), TAM2 (CD163+) cells, and regulatory T-cell-inducing immune cells (CD80+), revealed a negative impact of these cells on disease recurrence, similar to CAFs. This finding indicates that, in terms of prognosis, the assessment of the intensity of the overall TME-related inflammatory infiltrate should always be followed by assessment of the density of specific protumorigenic components.

It has been recently shown that signals from cancer cells can be transmitted to the inflammatory cells via exosomes [30]. Exosomes can exert an inhibiting effect on the function of T cells and on the differentiation and maturation of antigen-presenting cells from their precursor cells. In addition, they are able to increase the number and/or enhance the activity of immunosuppressive/protumorigenic cells, including myeloid-derived suppressor cells and regulatory T cells, resulting in suppression of the immune surveillance.

The present in vitro myoma and collagen assays showed a high expression of exosomal-associated markers (i.e., TSG101, TGF-*β*) in the HSC-3 cells and, to a lesser extent, in the stromal cells. This is in accordance with a recent study, in which the cancer cells were shown to release TGF-*β*-coated exosomes into the microenvironment [22]. It can be assumed that exosomes exert their effects in both autocrine and paracrine routes. The secreted exosomes can be taken up by the cancer cells via the autocrine route where TGF-*β* activates the Akt-Smad cascade and induces many gene transcription sites, including the gene responsible for the expression of *α*-SMA [22]. Functional and morphological alterations within the original epithelial cancer cells may ultimately occur. Indeed, our in vitro model enabled us to demonstrate the presence of cells surrounding the periphery of some invading tumor islands that showed remnants of cytokeratin expression, indicating their epithelial origin. It concomitantly revealed that they were predominantly twist positive, implicating these cells in the EMT process. Furthermore, the availability of serial sections enabled us to conclude that these positively double-immunostained cytokeratin and twist cells coincided with spindle-shaped *α*-SMA-positive cells. Identification of the HSC-3 cells assumed to have undergone EMT at the margins of the invasive tumor in the myoma model can be explained in terms of these cells being subject to microenvironmental stimuli distinct from those received by cancer cells located in the cores of the tumor [11]. Moreover, it can be assumed that the cancer cells do not pass through a complete EMT process, but rather only a partial one, thereby acquiring new mesenchymal traits while continuing to express residual epithelial traits [11]. This was shown in the present study, where the peripherally positioned, spindle cells exhibited twist and traits of cytokeratin. Importantly, these findings were restricted to the myoma model and they were absent in the collagen assay, emphasizing the superiority and uniqueness of the former to create conditions that mimic the natural TME.

TGF-*β*-coated exosomes are able to induce the transformation of the stromal cells to *α*-SMA-expressing cells [22] therefore constituting the paracrine route of action of the exosomes. Our in vitro study showed that HSC-3-derived exosomes probably induced the expression of *α*-SMA in the CaDEC cells, which subsequently resembled CAF-like cells, both morphologically (spindle-shaped) and spatially (subtle concentric cells surrounding the tumor nests/clusters). Our serial sections showed that the *α*-SMA-positive cells in the HSC-3-CaDEC cocultures were usually twist positive and CK negative. Moreover, the paracrine route of exosomal TGF-*β* can also be related to the modulation of the extracellular matrix and emergence of fibrotic tissue that always accompanied the invading tumor islands and that separated them from the bundles of smooth muscles of the myoma, possibly indicating that exosomal TGF-*β* can regulate fibrogenesis in order to facilitate tumor migration and invasion [31, 32]. In a recent review, it has been pointed out that the degree of the rigidity of the extracellular matrix is associated with the mechanical stress and interstitial flow in the tumor stroma, which is used by the CAFs as guidance tracks to direct cancer cell migration [33]. The extracellular matrix rigidity together with an impaired vascular and lymphatic network contribute to an increase in the interstitial fluid pressure that ultimately results in a reduced delivery of chemotherapeutics [33].

The myoma model also enabled us to show high expressions of cytokines, such as IL-1a, IL-1R1, IL-8, and NF-κB, which are all known to have reciprocal interactions. IL-1 can induce expression of NF-κB through IL-8, NF-κB induces expression of IL-1a, and IL-1R1 serves as the receptor through which NF-κB activates its downward cascade of intracellular effects. Altogether, these cytokines have the potential to promote tumor proliferation, growth, and invasion [24, 25, 28, 29, 33]. For example, the strong expression of CXCR4 that we found in the HSC-3 cells could have been induced by stromal TGF-*β*, the latter being necessary for the continued expression of CXCR4 [13]. Expression of CXCR4 in cancer cells generally activates the Akt-signaling pathways that are responsible for cellular invasion and proliferation. Interestingly, the expression of CXCR4 within the cancer cells was shown to also be induced by NF-κB [13]. Our in vitro study demonstrated strong expression of both CXCR4 and NF-κB within the HSC-3 cells. The existence of the cytokine crosstalk between cancer cells and CAFs had already been investigated in in vitro assays of oral SCC [34] and pancreatic ductal adenocarcinoma (PDAC) [29]. In those studies, cancer-derived IL-1a played a key role in inducing CAFs to release factors with functions in tumor migration and invasion (i.e., IL-1a, IL-8, IL-6, and others).

In summary, the clinicopathological correlations that emerged from this investigation highlighted significant associations between TME components (i.e., inflammatory protumorigenic cells and CAFs) and poor clinical outcomes in terms of recurrence and shortened survival. Our culturing of tongue cancer cells on a three-dimensional myoma organotypic model that mimics TME revealed the presence of cancer-derived exosomes, the EMT process, the acquisition of a CAF-like phenotype by fibroblasts, and reciprocal interrelations between different cytokines. Collectively, these results suggest the presence of molecular crosstalk between cancer cells and TME components in MT cancer. New therapeutic modalities to target this crosstalk should be developed in order to improve the prognosis of MT cancer patients.

## Supporting Information

**Table S1.** Antibodies, manufacturers, and laboratory procedures for immunostains of the inflammatory infiltrate cells, mediators, and cancer-associated fibroblasts in mobile tongue cancer of the human sections. The cancer-associated inflammatory cells were classified into protumorigenic (“bad”) and antitumorigenic (“good”) inflammation [9].
