# Supplementary material for: Molecular crosstalk between cancer cells and tumor microenvironment components suggests potential targets for new therapeutic approaches in mobile tongue cancer
Source: Cancer Med. 2012 Aug 16;1(2):128–40. doi: 10.1002/cam4.24 (PMC3544451; doi:10.1002/cam4.24)
Supplement: Supplementary file 1 [file cam40001-0128-SD1.doc]

**Supplemental Material: Table**: Antibodies, manufacturers and laboratory procedures for immunostains of the inflammatory infiltrate cells, mediators and cancer-associated fibroblasts in mobile tongue cancer of the human sections. The cancer-associated inflammatory cells were classified into pro-tumorigenic ("bad") and anti-tumorigenic ("good") inflammation (9).

| **Class of cells** | **Type of cells** | **Epitope/Clone/Manufacturer** | **Working procedure** |
| --- | --- | --- | --- |
|  | Macrophages, mature | CD68/ Clone kp1/  BioGenex, San Ramon, CA, USA | 1:30, 1h, room temp, Pronase K 0.1%, 37^o^C |
| Pro-tumorigenic inflammatory cells | Tumor-associated macrophages, M2 phenotype  Regulatory T cells (Tregs)  Treg-inducing immune cells | CD163/ clone k20-T/ Acris, Herford, Germany  Foxp3/ Clone mAbcam 22510/ Abcam,  Cambridge, UK  CD80/ clone 1G10/ Abcam, Cambridge, UK | 1:200, over night, citrate buffer pH6, pressure cooker  1:50, 1h, room temp, pH9, EDTA, PC  1:50, over night, citrate buffer pH6, pressure cooker |
| Anti-tumorigenic inflammatory cells | T cells, all | CD3/ polyclonal/  Zymed, San Francisco, CA, USA | Ready to use, 1h, room temp, pH9, EDTA, PC |
|  | T helper | CD4/ Clone ZT-17/  Zymed, San Francisco, CA, USA | Ready to use, 1h, room temp, pH9, EDTA, PC |
|  | T cytotoxic | CD8/ polyclonal/  Zymed, San Francisco, CA, USA | Ready to use, 1h, room temp, pH9, EDTA, PC |
|  | B lymphocytes | CD20/ Clone L26/  Dako, Glostrup, Denmark | 1:100, 1h, room temp, pH6 MW for 10 min |
|  | Plasma cells | CD138/ Clone B-B4/  Serotec, Kidlington, Oxford, UK | 1:100, 1h, room temp, pH9, EDTA, PC |
| Immune control | NF-κB | Polyclonal/ Alexis Biochemicals  San Diego, CA, USA | 1:500, over night, 4^o^C, pH6, citrate buffer,  PC |
| Other TME cellular component | CAFs | α-SMA / clone 1A4/ Dako A/S, Denmark | 1:100, 60 min, room temperature, citrate buffer, pH 6,  microwave at 92^o^C |
